# Stress and career aspirations: a longitudinal study with medical students

**DOI:** 10.3389/fpsyg.2024.1449111

**Published:** 2024-10-04

**Authors:** Clara Picker-Roesch, Jessica Lang

**Affiliations:** Medical Faculty, Institute for Occupational, Social and Environmental Medicine, RWTH Aachen University, Aachen, Germany

**Keywords:** generalized estimating equation (GEE), medical students, academic stress, career goals, gender

## Abstract

**Background:**

The psychological well-being of medical students has often been studied in relation to various factors and typically exhibits poorer scores than comparable student groups. This diminished well-being can have far-reaching implications, like impact on career or academic engagement. While there are some studies on specialty choice or academic performance few studies have examined the effects of stress at the beginning of medical school on career ambitions and the associated interest in research. Given the anticipated future shortage of healthcare professionals and the goal of gender equality in higher positions, it is crucial to examine this relationship as early as medical education. The question arises whether medical education and the demands of studying influence the career ambitions of different student groups.

**Method:**

As part of a longitudinal survey, students from a medical cohort are surveyed over three consecutive years. A total of 297 students participated in the surveys, of which 66.4% were women. Stress experiences during medical school and career aspirations are assessed and analyzed using Generalized Estimating Equation (GEE) modeling.

**Results:**

The results indicate that early academic stress is a negative predictor of the career ambitions of medical students (*B* = 1.637; *p* = 0.009). Also, female students report lower career goals than male students (*B* = 1.168; *p* = 0.018). In addition, gender moderates the impact of stress on career ambitions (*p* = 0.046). Finally, stress levels are highest when starting the medical program (*p* = 0.022) and decrease over time.

**Discussion:**

Stress during the initial medical training impedes students to pursue higher career ambitions. Future research as well as medical programs need to elaborate more on and consider structural stress prevention interventions in order to promote well-being and strengthen also scientific career goals at an early stage, specifically for female students.

## Introduction

‘The Positive Psychology Substance: The good life—what it is to be healthy and sane, and what humans choose to pursue when they are not suffering or oppressed’ Seligman (2019).

Seligman’s original definition of Positive Psychology encourages us to consider how external circumstances impact the human pursuit of fulfilment and well-being. When individuals are free from pressure and suffering, they can more clearly recognize and pursue their true desires and goals. But what happens when they are constrained by stress and burdens? Do their desires and goals change as supposed by Seligman?

These questions are particularly pertinent to medical students, a group that experiences significant stress. They must navigate numerous challenges in balancing their studies with the pursuit of professional goals, often leading to elevated stress levels and poor mental health (Dyrbye et al., 2006; Fares et al., 2016). Despite women making up the majority of medical students, they remain underrepresented in high-level medical positions (Deutscher Ärztinnenbund, 2022). Understanding gender-specific factors influencing career ambitions in medical education is crucial for addressing this disparity. This study explores how study-related stress influences career ambitions, with a particular focus on gender differences.

### Background on study-related stress

The mental health of medical students has consistently been shown to be poorer than that of the general population and students in other disciplines (Ashouri and Rasekhi, 2015; Dyrbye et al., 2006; Wilkinson, 2023). Academic stress in this group is particularly pronounced, driven by a demanding curriculum, heavy workload, high competition, and insufficient time for personal life (Imran et al., 2016; Radcliffe and Lester, 2003). Added to this are the effects of the Covid-19 pandemic, which have led to stress for students in particular (O'Byrne et al., 2021; Peng et al., 2023). To understand the underlying mechanisms of these correlations the present study builds on Self-Determination Theory (SDT) (Deci and Ryan, 2000). SDT posits that individuals have innate psychological needs for autonomy, competence, and relatedness, which are critical for motivation and well-being (Behzadnia and FatahModares, 2020; Deci and Ryan, 2000). Increased stress and low fulfillment of needs can therefore lead to lower motivation during studies (Barbayannis et al., 2022; Li and Hasson, 2020). Research on stress progression during medical education shows mixed findings, with some studies highlighting increased stress at the start of medical school (Jafari et al., 2012), while others report no alteration (Franzen et al., 2021) or the poorest mental health in specific years (Shadid et al., 2020), depending on the country (Jafari et al., 2012). These variations may be influenced by differences in curricula, degree structures, and examination systems.

Gender differences are particularly significant, with female medical students reporting higher stress levels and a greater prevalence of anxiety disorders compared to their male counterparts (Franzen et al., 2021; Jestin et al., 2023). According to SDT women may face more substantial barriers in fulfilling their psychological needs, especially in terms of relatedness, as Spoon et al. (2023), for example, has shown that women are more likely to leave science due to a poor working environment than men. Ameer et al. (2022) further support this by revealing that women experience higher levels of perceived academic stress compared to men, which may indicate that stress more significantly impacts women’s ability to fulfill their psychological needs and aspirations. However, most studies on stress among medical students primarily focus on stress effects on mental health conditions, such as depression, while positive aspects related to stress prevention or career-related topics are not examined in detail or with extensive samples (Amanvermez et al., 2023; Mirza et al., 2021; Ruzhenkova et al., 2018).

### Background on career aspirations

Research on the career aspirations of medical students has primarily focused on specialty selection, revealing gender differences in both choosing and changing subspecialties (Byrnes et al., 2020; Compton et al., 2008; Jacob et al., 2023; Larsen et al., 2021; Levaillant et al., 2020). Fewer studies have explored career position choices, though recent findings from Germany indicate a decline in aspirations for higher positions as studies progress (Jacob et al., 2023). This decline may be attributed to the dual burden of combining research with clinical duties, particularly in university hospitals where this combination is often required (Cody and Rosales, 2021; Sorg et al., 2016).Over time, this burden, exacerbated by parenthood, has led to reduced research activities, especially among women who prioritize work-life balance (Buddeberg-Fischer et al., 2010; Frank et al., 2019).

Despite women comprising the majority of medical students, they hold significantly fewer leadership positions in clinics and university hospitals (Deutscher Ärztinnenbund, 2022; Richter et al., 2020). According to SDT, these disparities may result from differing needs for autonomy and relatedness, with women possibly prioritizing a supportive work environment and work-life balance over high-stress roles (Spoon et al., 2023). Research on SDT also indicates that lower stress and better coping with challenges are linked to higher autonomous functioning, greater mindfulness, and a focus on intrinsic rather than extrinsic goals, which could explain the negative relationship between stress and career aspirations among students (Weinstein and Ryan, 2011). To complement this theoretical context, the Career-Construction Theory (Savickas, 2005) provides suitable approaches. CCT emphasizes that career choices are shaped by individuals’ experiences, values, interests, and abilities. It complements the SDT by suggesting that high stress levels might lead students to focus on immediate, short-term goals, like passing exams, that align more closely with their values and provide immediate psychological satisfaction (Urbanaviciute et al., 2019). Potentially this is steering them away from high-level career aspirations (Sharma, 2014) and leading to gender-specific career outcomes. Additionally, resilience and career decision-making difficulties among students are negatively correlated, indicating that lower levels of stress during studies, in line with CCT, lead to clearer career goals (Pang et al., 2021).

Although career stages in medical practice have been studied, their development during undergraduate education, particularly longitudinal changes alongside specialization, remains underexplored. This study addresses this critical research gap by investigating how academic stress and gender influence early career aspirations among medical students. By examining stress trajectories and career goals over the first 3 years of medical school, this research uniquely integrates these factors, which are often studied in isolation. The findings aim to contribute to ongoing discussions on mental health and gender disparities in academia (Hall, 2023; Spoon et al., 2023), offering insights that support the development of students’ career aspirations.

### Hypotheses

Drawing from SDT (Deci and Ryan, 2000) and CCT (Savickas, 2005), the research investigates how academic stress impacts career aspirations, with a specific focus on gender differences during the first 3 years of medical education. Previous studies have shown that high academic stress negatively affects well-being and motivation, potentially diminishing long-term career ambitions (Barbayannis et al., 2022; Li and Hasson, 2020). Within the framework of SDT, stress undermines psychological needs, leading students to prioritize more attainable, short-term goals over challenging, long-term aspiration (Ameer et al., 2022; Deci and Ryan, 2000). Similarly with CCT it can be suggested that high stress impairs active career planning, driving students to seek quicker satisfaction of psychological needs through less ambitious career goals (Savickas, 2005). Therefore, the first hypothesis is:

*Hypothesis 1: Academic stress negatively correlates with career goals.*

As mentioned above, women tended to report higher academic stress during medical education (Jestin et al., 2023). Additionally, they reported to have lower motivation for high positions after graduation, sought more work-life balance, and were significantly underrepresented in leadership positions in the medical field (Buddeberg-Fischer et al., 2010; Lefevre et al., 2010; Richter et al., 2020). Thus the following hypothesis can be stated:

*Hypothesis 2a: Gender impacts career goals, with female students reporting lower career aspirations than their male peers.*

Given evidence that men and women may respond differently to stress (Matud, 2004; Tamres et al., 2002) and women experiencing other fulfilment of their psychological needs (Ameer et al., 2022; Spoon et al., 2023), it is important to explore how gender moderates the relationship between stress and career aspirations. Higher stress levels, particularly among women, may lead to differing approaches to goal setting:

*Hypothesis 2b: Gender moderates the negative association between academic stress levels and career goals, such that women are more strongly influenced by stress in their career aspirations compared to men.*

Finally, considering that stress trajectories can vary throughout medical education, with some studies indicating fluctuations in stress levels during different stages of the program (Franzen et al., 2021; Jafari et al., 2012; Shadid et al., 2020), this study hypothesizes the following:

*Hypothesis 3*: *Over the course of 3 years, there is a corresponding decrease in perceived stress levels.*

## Methods and measures

### Procedure

To address the formulated hypotheses, a longitudinal design was implemented. A cohort of medical students was surveyed at the beginning of each winter semester in the 1st, 3rd, and 5th semesters using paper-pencil questionnaires in a within-subject design. The surveys were conducted prior to a general progress test, ensuring that all students in the cohort were surveyed simultaneously. The students were queried about their career aspirations, stress experiences, and various demographic data. To match the data from the different years, students created an individual code consisting of numbers and letters, which allowed for longitudinal tracking without revealing any personal information. The study was approved by the local ethics committee (EK 23–112), and participants provided informed consent before completing the surveys.

### Participants

The cohort consisted of 297 medical students, resulting in 577 cases across the three time points. In the first semester, the response rate was 72.7% (216 cases), in the third semester 50% (148 cases), and in the fifth semester 71.7% (213 cases). Of the 577 completed questionnaires, 66.4% were filled out by women and 33.6% by men. Complete data for all three time points were available for 87 individuals (29.3%), and two time points were available for 106 individuals (35.7%). The average age of participants was 21.3 years. In Germany, admission to medical school is primarily determined by achieving excellent grades in the Abitur (secondary school diploma). For the first semester, 42% of students were admitted through university-specific selection processes, which primarily consider the Abitur grade, a medical aptitude test, and, in some cases, relevant vocational training. One-third of the students were admitted based on the top Abitur grade quota, while the remaining students entered through quotas for rural doctor placements, waiting times, or other special criteria. Detailed sample descriptions per year, as well as information on career aspirations, are provided in Table 1.

**Table 1:** Participants description.

	T1	T2	T3	Overall
N	216	148	213	577
Age	19.8 (SD = 3.2)	21.9 (SD = 3.5)	22.3 (SD = 3.8)	21.3 (SD = 3.7)
Gender				
Men	78	40	76	194
Women	138	108	137	383
Highest career goal				
Employment in a practice	3	1	5	9, 1.8%
Specialist in a clinic	4	4	3	11, 2.2%
Private/group practice	37	41	52	130, 26.3%
Senior physician in a clinic	62	37	63	162, 32.7%
Chief physician/clinic director	46	14	31	91, 18.4%
Professor	25	32	35	92, 18.6%

### Measures

The same questionnaire was used at all three time points. In addition to demographic information such as age, gender, and semester of study, participants could indicate their current career aspirations with multiple responses. The scale ranged from six options (1–6): “Employment in a practice,” “Specialist in a clinic,” “Private/group practice,” “Senior physician in a clinic,” “Chief physician/clinic director,” to “Professor.” Additionally, there were options for “Other” and “Undecided.”

Perceived stress in medical school was measured using the Medical Student Stressor Questionnaire (MSSQ) (Yusoff and Rahim, 2010), which consists of six subscales and a total of 23 items (Yusoff et al., 2010). Stress caused by specific factors in medical school was rated on a 5-point Likert scale ranging from “Causing no stress at all” to “Causing severe stress.” The stressors are categorized into the following areas: academic-related, intrapersonal, and interpersonal-related, teaching and learning-related, social-related, drive and desire-related, and group activities-related. The Cronbach’s alpha for the overall scale is 0.899, indicating good reliability (Moosbrugger and Kelava, 2012).

### Statistical analysis

The data analysis was conducted using SPSS statistical software, version 29 (IBM Corp. Released, 2022). A total of 577 cases were analyzed, with 472 (81.8%) included in the final analysis. For the preparation of highest career goal data, the response options “Other” and “Undecided” were not considered for the ordinal scale. The highest hierarchical career aspiration was used as the data point for analysis. For stress experience, a mean score across the entire MSSQ scale was calculated, following the guidelines of the authors (Yusoff and Rahim, 2010). Gender was treated as a binary variable, as only the options “male” and “female” were selected.

In addition to descriptive analyses of the individual time points, a linear model was tested to examine the hypotheses. Stress experience and gender served as predictors, and “highest career goal” was the multinomial dependent variable. The model accounts for the main effects of stress experience (MSSQ, Hypothesis 1) and gender (Hypothesis 2a), as well as the interaction between these variables (Hypothesis 2b). Including the interaction allows for the investigation of the moderating influence of gender on the relationship between stress and career aspirations. To test Hypothesis 3, the dependent variable was stress perception, and the time of measurement, i.e., the respective semester, was treated as a categorical factor. Given the repeated measures and the ordinal nature of the dependent variable “career goal,” a robust Generalized Estimating Equations (GEE) model with an ordinal logistic model type was employed. A GEE model is better suited for data analysis dealing with correlated data, and provides more robust and flexible estimation methods than ANOVA or cross-lagged panel designs. Furthermore, GEE offers a more versatile approach particularly in longitudinal study designs (e.g., Melo et al., 2022).

## Results

**Figure 1:**
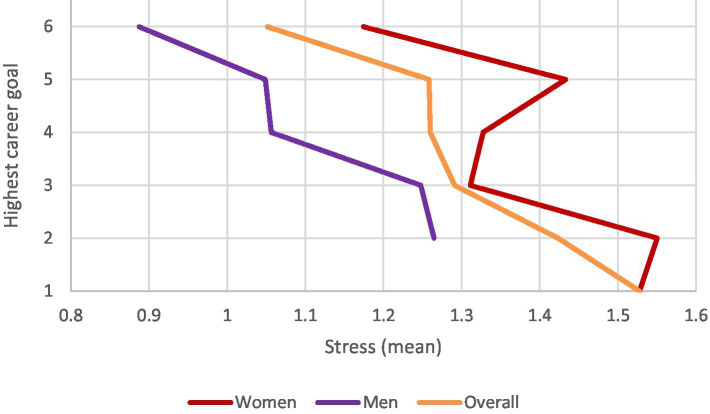
Correlation of stress levels with each highest career goals by gender and the overall sample.

**Figure 2:**
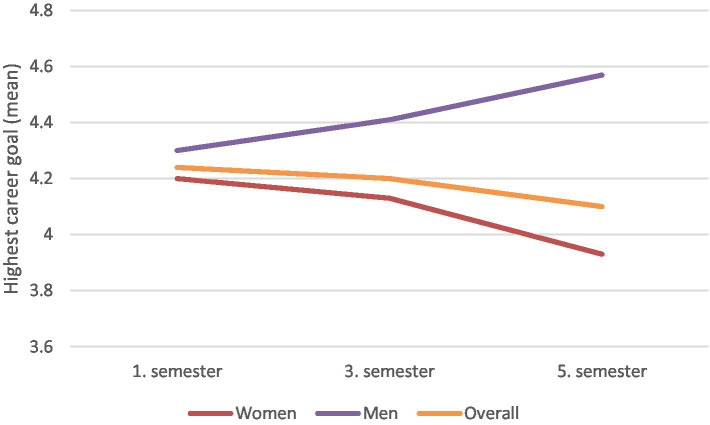
Highest career goal during the first three study years by gender and the overall sample.

**Figure 3:**
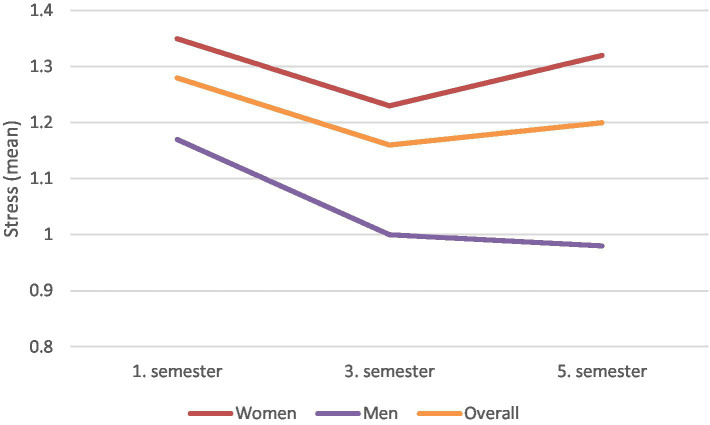
Stress levels during the first three study years by gender and the overall sample.

To test Hypotheses 1–3, descriptive values were calculated (Figures 1–3, Table 2) and the described GEE model was implemented. The estimates of the coefficients, standard errors, 95% confidence intervals, Wald chi-square statistics, and associated one-sided *p*-values are presented in Tables 3, 4.

**Table 2:** Correlation table of study variables.

Variables	M	SD	1	2	3	4
1. Gender	0.66	0.473	–			
2. Semester	2.99	1.726	0.004	–		
3. Highest career goal	4.19	1.186	−0.14**	−0.032	–	
4. Stress	1.22	0.545	0.215**	−0.067	−0.162**	–

** Correlation is significant at the 0.01 level (2-tailed). Gender coding: 0 = male and 1 = female.

**Table 3:** GEE model with stress and gender predicting highest career goal.

Variables	B	Std. error	95% confidence interval	Wald χ^2^	*p*-value
Threshold					
[Highest career goal = 1, 00]	−4.156	0.612	[−5.356, −2.956]	46.069	<0.001
[Highest career goal = 2, 00]	−3.441	0.490	[−4.402, −2.479]	49.208	<0.001
[Highest career goal = 3, 00]	−1.044	0.394	[−1.816, −0.271]	7.012	0.004
[Highest career goal = 4, 00]	0.342	0.390	[−0.422, 1.105]	0.769	0.190
[Highest career goal = 5, 00]	1.315	0.411	[0.510, 2.120]	11.490	<0.001
Stress	−1.637	0.685	[−2.980, −0.293]	5.696	0.009
Gender					
[Gender = 0 = male]	1.168	0.554	[0.082, 2.254]	4.444	0.018
[Gender = 1 = female]	0a	.			
Interaction: gender x stress	0.695	0.412	[−0.112, 1.503]	2.848	0.046

GEE: Generalized Estimating Equations. a = Reference category. *P*-values for one-sided hypothesis testing.

**Table 4:** GEE model with study semester predicting stress.

Variables	B	Std. error	95% confidence interval	Wald χ^2^	*p*-value
Intercept	1.197	0.0393	[1.120, 1.274]	927.324	<0.001
[semester = 1]	0.085	0.0421	[0.002, 0.167]	4.059	0.022
[semester = 3]	−0.033	0.0463	[−0.124, 0.058]	0.510	0.238
[semester = 5]	0a				

GEE: Generalized Estimating Equations. a = Reference category. *P*-values for one-sided hypothesis testing.

To test Hypothesis 1, Table 2 provides the mean (M), standard deviation (SD), and Pearson correlation coefficients for all study variables. Notably, the data show a significant negative correlation between stress and the highest career goal. This finding is visualized in Figure 1, which illustrates the average stress levels associated with different career goals. It’s important to consider the overrepresentation of women in the sample when interpreting these descriptive results. However, Figure 1 reveals that women generally report higher stress levels and that, overall, there is a negative correlation: lower stress is associated with higher career aspirations. Additionally, the GEE confirmed that stress levels serve as a significant and negative predictor of the highest career goal among medical students (*B* = 1.637, *p* = 0.009). The thresholds were significant and negative for all categories of the final highest career goal, except for the goal of senior physician. This suggests that the more students reported experiencing study-related stress, the less likely they were to strive for higher career goals, supporting Hypothesis 1.

To address Hypothesis 2a, we first examined the descriptive data in Table 2 and the GEE in Table 3, which demonstrate a significant gender effect on career goal decisions, with men being more likely than women to aspire to higher career goals (*B* = 1.168, *p* = 0.018). These results were visualized by Figure 2, which illustrates that the development of the highest career goals over 3 years, differentiated by gender. The figure shows that, over the course of their studies, men tend to pursue higher career goals, while women aim for comparatively lower goals. Thus, Hypothesis 2a is supported.

For testing the moderation effect of gender on the relationship between stress and career goals in hypothesis 2b, the GEE analysis resulted in a significant interaction effect (*B* = 0.695, *p* = 0.046). The interaction can descriptively be observed in Figure 1. The direction of the interaction effect indicated that the negative impact of stress on career goals was more pronounced in female students compared to male students. Therefore, Hypothesis 2b is supported.

Hypothesis 3 was further tested using a GEE model, as detailed in the ‘Statistical Analysis’ section, with the results presented in Table 4. The analysis revealed a significant effect of the semester on stress levels (Wald χ^2^ = 6.340, *p* = 0.022). Specifically, the first measurement point at the beginning of the studies showed higher values than the other two measurement points, supporting Hypothesis 3. Figure 3 illustrates the development of academic stress over time. It shows that stress levels decrease for the overall sample from the first to the third semester, but rise again for women in the fifth semester.

## Discussion

The overarching aim of this study was to identify the influence of study related stress and gender on career decisions of medical students. This knowledge can enable better support and encouragement especially for female student to strive for higher career goals. A cohort of medical students in a German medical school was surveyed at the beginning of their first 3 years of study. As expected, analyses demonstrate a negative correlation between stress levels and career goals (Table 3), indicating that students with lower stress tend to pursue higher career aspirations. Additionally, the data reveal that men aim for higher career goals compared to women (Figure 2, Table 3) whereby the goals of men increase and those of women decrease over time. Also, the interaction between gender and stress showed a significant influence on career goals (Figure 1, Table 3). Thus, there is a moderating effect of gender on the relationship between stress and career goals, with the negative effect being greater in women. The examination of the influence of time on stress (Figure 3, Table 4) showed that it differs significantly between the three time points, with particularly high stress levels at the first measurement point.

### Interpretation

The results of this study validate all four hypotheses and align with the explanatory models of SDT and CCT. As anticipated, lower stress levels are associated with higher career goals, consistent with the principles and findings of both SDT (Deci and Ryan, 2000; Weinstein and Ryan, 2011) and CCT (Pang et al., 2021; Savickas, 2005). High academic stress, characterized by high expectations, external pressure, and diminished autonomy, leads medical students to pursue less ambitious career paths early in their studies. In fact, major stress reported by medical students was caused by exams and insufficient time for reviewing the material. This findings underscores the importance of autonomy and motivation as emphasized by SDT (Deci and Ryan, 2000) and the career adaptability central to CCT (Savickas, 2005).

Neufeld et al. (2020) further elucidates these dynamics by demonstrating that increased mindfulness and resilience, alongside the satisfaction of basic psychological needs, are associated with lower perceived stress. Conversely, the frustration of these needs correlates with higher stress levels, according to SDT. Neufeld et al.’s (2020) findings suggest that when students’ basic psychological needs are thwarted, the stress-buffering effects of individual inner coping resources are compromised. This aligns with our observation that female students experience higher stress and reduced need satisfaction, particularly in advanced academic years.

Figures 1–3 illustrate that while both male and female medial students initially set high career goals, women’s aspirations decline under stress, whereas men’s ambitions often rise. The moderating effect of gender, reveals that women may adjust their career goals as a coping mechanism to manage increased stress during the course of their studies (Ameer et al., 2022; Spoon et al., 2023). This adjustment may help explain the underrepresentation of women in leadership positions within the medical sector among physicians (Deutscher Ärztinnenbund, 2022). Haushofer et al. (2021) corroborate this by demonstrating that high stress levels are generally associated with lower, short-term career goals, reinforcing the broad impact of stress on career trajectories.

Querido et al. (2016) identified five factors influencing career goals, including medical school characteristics, student characteristics, student values, career needs, and perceptions of specialty characteristics. These factors align with our findings on how stress and gender affect career aspirations. For example, men’s greater propensity to pursue challenging career goals is linked to differing social expectations and personal values (Dicke et al., 2019), while women often prioritize family, work-life balance, and workplace climate (Buchinger et al., 2022; Heiligers, 2012; Spoon et al., 2023). Additionally, men in medical education typically receive more support, whereas women face a lack of role models (Kramer et al., 2021).

Further, Matud (2004) and Tamres et al. (2002) highlight gender differences in stress responses and coping strategies. Our findings are consistent with these studies, showing that women are more affected by academic stress and tend to lower their career goals as a coping mechanism. Similarly, Cahlíková et al. (2020) found that women’s susceptibility to stress often results in an early reduction of career ambitions, aligning with the principles of CCT.

The temporal analysis reveals that students experience higher stress at the beginning of their medical education compared to later stages. This observation aligns with findings from Jafari et al. (2012) and Shadid et al. (2020), who also reported high stress levels at the onset of medical studies. The rise in stress levels during the fifth semester (Figure 3) may be linked to the upcoming preclinical exams in the 6th semester, which are crucial for advancing to the clinical phase of medical studies in Germany. Rayner and Papakonstantinou (2020) suggest that the balance between intrinsic and extrinsic motivation evolves over time, with intrinsic motivation generally increasing. This progression, influenced by the degree of autonomy (SDT), could positively impact students’ intrinsic motivation and long-term career planning if enhanced in medical curricula.

### Limitations

Several limitations may have influenced our study findings. The smaller sample size in the third year, with only 87 participants consistently matched across all measurement points, potentially limited the statistical power and generalizability of the results. A more comprehensive, long-term data collection would have been preferable to better understand the impact of stress and career goals throughout the entire medical education and early career phases. Future research should extend these investigations over a longer period to provide a more nuanced understanding.

The focus on only three influential factors, i.e., stress, gender, and semester may have overlooked other influential variables. Including additional factors, such as study conditions or perceptions of autonomy, could yield more detailed insights into the parameters affecting career goals. The present study solely relied on self-reported data which can fluctuate within the context of education and study structure. The consideration of more objective chronic stress parameters like hair cortisol concentration (Quinete et al., 2015) could undermine the relevance of stress-induced effects.

The career stage scale used in this study, developed by the researchers, may lack clarity regarding the hierarchy of career stages and could be assessed differently, especially at the beginning of the studies. Future research should evaluate this scale or establish a ranking system based on students’ evaluations of career stage value. Finally, the study’s focus on a single cohort from one university limits the generalizability of the findings. Conducting a longitudinal analysis across multiple institutions would be beneficial to address these limitations and enhance the understanding of stress and career development in medical education.

### Implications and future studies

The results of this study have important implications for medical education practice and policy. To address the shortage of skilled professionals and promote gender equality, changes in medical education are necessary. In line with Hill et al. (2018) both personal and system-based factors can be adjusted, with an emphasis on system-based factors, as suggested by Schmidt et al. (2015). A focus on structural prevention aligns with these findings, where academic stress can be mitigated by altering curricula and introducing more flexible learning conditions from the start (Slavin et al., 2014).This approach can reduce pre-existing stress and prevent new stress from building. Reducing academic stress may lead to higher career aspirations, as well as support long-term student well-being and motivation. Early integration of these strategies into the curriculum ties into principles of positive psychology, offering students early career support and enhancing their overall development.

Additionally, targeted programs should be developed to address the specific needs of female medical students. Alongside a revised curriculum, female mentors could support students from the beginning, serving as role models and demonstrating work-life balance. To further complement these efforts, behavioral prevention interventions such as stress management training should be offered to all students, providing additional support (Frei et al., 2010; Shiralkar et al., 2013). Furthermore, specific programs or interventions based on the CCT can be offered to directly promote career choice (Wang and Li, 2024).

Future studies should explore additional factors influencing career goals. Firstly, examining study conditions and curricula, as well as students’ personality and health factors, could be valuable. Secondly, comparisons with other universities and countries would be worthwhile to assess cultural influences and generalize the results. Longitudinal studies following students throughout their studies and into their professional careers could reveal further factors, including those dependent on time, that are significant for medical professionals. Additionally, investigating the impact of practical experiences with physicians and clinical placements would be interesting, as these experiences could strongly influence students’ career decisions. Here, a focus on gender differences would also be relevant, taking into account gender research and development.

## Conclusion

In conclusion, this study offers important insights into the factors shaping the career goals of medical students, particularly the significant roles of stress and gender. High academic stress is found to negatively affect career aspirations, while gender differences reveal that men tend to pursue more ambitious goals than women.

Using CCT and SDT frameworks, it is evident that early stress limits long-term ambitions, especially for women, emphasizing the need for structural changes and gender-specific support in medical education. To address these challenges, improving curricula, adapting study conditions, and implementing equal-opportunity programs, such as mentorships for female students, are essential. Additionally, in line with positive psychology, behavioral prevention interventions can enhance students’ well-being, motivation, and career goals from the start, promoting gender equality in leadership over time.

Future research should expand on these findings by examining additional factors, such as study conditions and personality traits, through diverse and longitudinal studies. A comprehensive approach that considers both structural and behavioral factors will strengthen medical education, promote gender equality, and alleviate the shortage of skilled professionals.

## Data Availability

The raw data supporting the conclusions of this article will be made available by the authors, without undue reservation.
